# Notes on the Advancement of the Ocular Muscles1Read before the Section on Ophthalmology, Otology, Rhinology and Laryngology, Buffalo Academy of Medicine, April 16, 1900.

**Published:** 1900-07

**Authors:** Benjamin H. Grove

**Affiliations:** Buffalo, N. Y., Surgeon to the Charity Eye, Ear and Throat Hospital


### NOTES ON THE ADVANCEMENT OF THE OCULAR MUSCLES.[1]

By BENJAMIN H. GROVE, M. D., Buffalo, N. Y.,
Surgeon to the Charity Eye, Ear and Throat Hospital.

THERE is now something of a fad of eye-muscle cutting. I thoroughly believe in the conservative practice of tenotomy of the ocular muscles yet the tendency is to go to the extreme. Everyone who desires to be conscientious in the matter, I mean by that, do *more* generally than just snip or button-hole the tendon and has had a large experience in operating can expect at times to over do his intentions. This requires the operator who makes tenotomies to be as equally able to catch up or advance a muscle. I have known those who do exclusive muscle work to over correct, and it has been the writer's experience to have caused homonymous diplopia when operating on the external rectus for an exophoria or turning of the eyes outward as well as in cases of convergence. Of course, such errors when noticed twenty-four to thirty-six hours after the tenotomy are easily corrected. However, there is no absolute method to be applied in the way of exact adjustment, so in many cases one must feel his way, as it were, and divide the operation between the eyes and, even then, make partial tenotomies more or less complete, depending upon the amount of heterophoria. This question is so complicated that many operators of experience hesitate about making tenotomies and the writer can testify that time has rather inclined him to be more cautious than formerly in this respect Probably this applies more especially to the tenotomy of the internal rectus or recti for convergent squint. This is not the simple matter that in our college days we were taught to believe. Much can be done with atropine and properly adjusted glasses, especially in the case of children, and perfect adjustment by tenotomies alone should not be made before the patient is ten to fifteen years old. The writer remem-

1. Read before the Section on Ophthalmology, Otology, Rhinology and Laryngology, Buffalo Academy of Medicine, April 16, 1900.

bers the wordy conflicts carried on by Von Arlt and Stellwag, of Vienna, in regard to the age for operating. Von Arlt was inclined to operate at an early age and Stellwag used to tell us he had to patch up Von Arlt's cases in after years. The four recti muscles are as occasion requires advanced, but it is to the operation for the advancement of the internal rectus that I wish especially to call your attention. Such advancement is called for in three different groups of cases. First, depending upon an injury or an operation, as I said, causing divergent squint, depending upon such injury or an over correction for convergent squint: Second, it may be paralysis of the internal or external rectus or, in the third place, from loss of sight or greatly impaired vision in one eye, producing dissociation.

In the first and second condition the muscles retain a degree of contractility, while in the case of paralysis there is often none at all. My attention has been forcibly called to this operation by meeting half a dozen cases during the past four months. I present here a photograph of an interesting case which I operated on a short time ago; one taken before and the other three weeks after the operation. The result of this operation, as you will notice, is excellent. This patient was twenty-five years old. She was operated on by an oculist of good reputation in this city for convergent squint some years ago. Divergent squint resulted and three or four times under chloroform another operator apparently attempted advancement. When I saw her there was divergence of one-third of an inch and the left eye could not be turned in at all. I remember this operation formerly seemed to me somewhat of a bugaboo. In fact, I felt more confident in making an extraction for cataract than in promising a successful advancement. Special toothed forceps, among other instruments, have been designed, and the books speak of severe reactions following the operation, but neither of these conditions have I found necessary or likely to follow. Formerly I was accustomed to dissect the muscle and grasp it with this special forceps, always fearful that it would slip away, but now I advance the muscle together with the oculo-orbital fascia or capsule of Tenon. The essential steps or conditions of the operation which I have found most practical are:

First, a vertical stitch of black silk, number 4 to 6, passed close to the cornea, its middle third, on the nasal side, through conjunctiva and dipping into the sclerotic tissue. This stitch is tied and with needle is laid over forehead and cheek.

Second, with strong fixation forceps, with toothed ends grasping the conjunctiva and underlying tissue, about one-half inch from the cornea on the nasal side, make a large vertical incision and then at each end a horizontal incision.

Third, make a complete tenotomy of the external rectus of the eyeball which you are manipulating, and if desirable of the other eye.

Fourth, now pass the needle of what we may call the corneal stitch from within outwards far back through mass of internal rectus muscle, capsule and conjunctiva. Then pass two stitches of white silk from without inwards through the same mass of tissue, one above and the other below, and pass the one above under the conjunctiva and into the scleral tissue, close to the upper border of the cornea and up to a line drawn vertically through the cornea. Then pass the other suture in the same manner below the cornea. Cut off redundant tissue and tie tightly, being sure to turn eyeball somewhat inwards.

Fifth, next fasten sutures through the stump of the detached external rectus or recti and asking patient to converge, strongly pass the sutures over the end of the nose and cover with plaster and collodion. Bandage both eyes securely and do not remove bandage for two or three days. Bathe eyelids and rebandage for two more days, but do not remove the stitches until five to seven days after the operation. The needles should be curved on the flat, and should have extra sharp points.

